# The effects of dog management on *Echinococcus* spp. prevalence in villages on the eastern Tibetan Plateau, China

**DOI:** 10.1186/s13071-020-04082-6

**Published:** 2020-04-21

**Authors:** Xiaodong Weng, Zhiqiang Mu, Xu Wei, Xu Wang, Qingqiu Zuo, Shuo Ma, Youzhong Ding, Xiaoming Wang, Weiping Wu, Philip S. Craig, Zhenghuan Wang

**Affiliations:** 1 School of Life Sciences, East China Normal University, Shanghai, China; 2 National Institute of Parasitic Diseases, Chinese Center for Disease Control and Prevention, Shanghai, China; 3 Shanghai Science and Technology Museum, Shanghai, China; 4 Joint Translational Science & Technology Research Institute, East China Normal University, Shanghai, China; 5 School of Environment and Life Sciences, University of Salford, Greater Manchester, UK; 6 Shanghai Key Laboratory of Urbanization and Ecological Restoration, East China Normal University, Shanghai, China

**Keywords:** Tibetan Plateau, Shiqu County, Dog management, Feces, Copro-PCR, Prevalence, *Echinococcus multilocularis*, *E. granulosus*, *E. shiquicus*

## Abstract

**Background:**

The pastoral area of the eastern Tibetan Plateau is highly endemic for human echinococcosis. Domestic dogs are the main definitive host for the transmission of both *Echinococcus granulosus* (*sensu lato*) and *E. multilocularis* to humans. To control the infection risks, a national-level canine echinococcosis prevention and control programme has been implemented since 2015 in Shiqu County, Ganze Tibetan Autonomous Prefecture, Sichuan, China. The objective of this study was to evaluate its effect on *Echinococcus* spp. prevalence in dogs.

**Methods:**

We surveyed 69 households with 84 owned dogs, for dog fecal samples and dog keeping information in the villages of Rizha and Eduoma. A total of 105 dog fecal samples (75 from owned dogs and 30 unknown dog fecal samples) were collected between 2015–2017 to determine *Echinococcus* spp. prevalence using copro-PCR. Eight variables based on household surveys were included into a logistic regression model for significant risk factors to canine echinococcosis prevalence in dogs.

**Results:**

Between 2015–2017, the overall *Echinococcus* spp. copro-DNA prevalence decreased significantly in dogs from 51.2% (2015) to 20.0% (2017) in Rizha, and insignificantly from 11.5% (2016) to 4.3% (2017) in Eduoma. *Echinococcus multilocularis* was the most prevalent species continually copro-DNA detected during the entire study period, while *E. granulosus* was rare and not detected in 2017. *Echinococcus shiquicus* copro-DNA prevalence (a probable non-zoonotic wildlife species) was as high in dogs as that of *E. multilocularis*, although only detected in 2015 in Rizha. Unleashed dog feces were mainly collected in Rizha in 2015. Although 93.2% of owned dogs were leashed, and the monthly praziquantel dosing rate reached 97%, *E. multilocularis* infection could still be detected in 11.1% of owned dogs in 2017. Monthly deworming, leashing dogs 24 h per day, and the avoidance of dogs feeding on livestock viscera were significant measures to prevent canine echinococcosis infection in owned dogs.

**Conclusions:**

Carrying out a canine echinococcosis prevention and control programme can significantly decrease *Echinococcus* spp. prevalence. The potential contact between leashed dogs and wild small mammals is still a risk for re-infection of owned dogs with *E. multilocularis*. This study shows that the long-term application of regular dog treatment with praziquantel in the vast and remote echinococcosis endemic areas of the eastern Tibetan Plateau can reduce transmission in dogs but remains a challenging intervention.
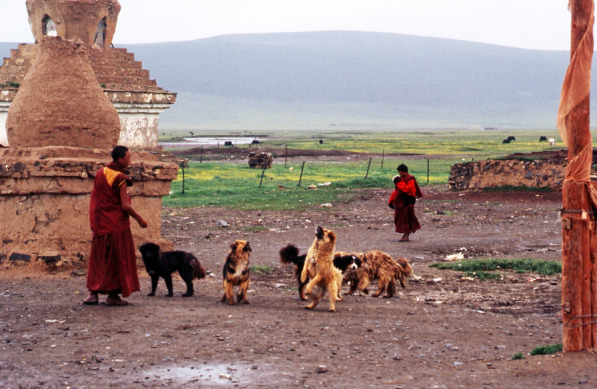

## Background

Echinococcosis is a potentially lethal and globally distributed zoonosis, caused by tapeworms from the genus *Echinococcus* [1]. Two forms of echinococcosis, cystic echinococcosis (CE) caused by infection with the metacestode of *E. granulosus* (*sensu lato*) and alveolar echinococcosis (AE) caused by infection with the metacestode of *E. multilocularis*, were found to be endemic in the pasture areas of western China threatening more than 50 million people [2]. AE is the most severe form of echinococcosis, with a mortality rate reaching 94% within 10 years without treatment [3]. About 91% of new cases of human AE reported worldwide occur in China [4, 5]. Echinococcosis has been listed as a critical endemic disease in China, and patients have been given free treatment from the Chinese national medical system since 2007 [6, 7].

Shiqu County, in Ganze Tibetan Autonomous Prefecture, Sichuan Province, China, is located in the pasture areas of the eastern Tibetan Plateau, has been reported to have the highest international prevalence rate of human echinococcosis, and is one of the most serious endemic regions of the world [8, 9]. Three *Echinococcus* species, *E. granulosus* (*s.l*.), *E. multilocularis* and *E. shiquicus*, coexist in this area. While *E. granulosus* is mainly transmitted between canids and livestock, *E. multilocularis* and *E. shiquicus* are mainly transmitted between canids and small mammals [10]. The dog is the only confirmed definitive host of both *E. multilocularis* and *E. granulosus* in Shiqu County [11, 12]. However, Boufana et al. [13] suspected that dogs may be also viable definitive hosts of *E. shiquicus* because a copro-DNA prevalence of 30% (6/20) was detected in fecal samples of owned dogs in Shiqu County, although unlike the other two *Echinococcus* species, *E. shiquicus* has not been reported currently as infecting humans. As a typical pastoral livestock husbandry county, pastoralism requirements and local Tibetan cultural traditions result in large numbers of owned and stray dogs to be kept in Shiqu County [8]. Because of their close relationships to local people, dogs are considered as the main risk to humans becoming infected with echinococcosis by ingesting *Echinococcus* eggs voided in dog feces [9, 11]. Therefore, methods to control the dog population size and decrease *Echinococcus* prevalence are critical, but a great challenge over many years for the control and prevention of echinococcosis in pastoral Tibetan communities [6, 7].

To provide better living conditions, the Chinese central government has started a settlement construction programme for pastoral Tibetan communities from 2004, causing a large expansion in the areas of original villages and towns. In addition, with better education opportunities and medical support, increased settlement of pastoral Tibetan families resulted in an increase in both owned and unowned (stray) dog populations, potentially increasing the risks of transmitting echinococcosis to humans. Therefore, the Chinese central government started a new pilot echinococcosis prevention and control project in Shiqu County in November 2015 [14]. As a crucial part of the project, dog management regulations included restricting the number of owned dogs to no more than two individuals per household, restricting and leashing owned dogs when staying in human settlements, controlling and decreasing the number of unowned dogs through fertility control, fostering, or humane euthanasia, and most importantly deworming registered dogs monthly with praziquantel and burying or burning voided fecal matter.

To evaluate the effects of the dog management regulations, we tested *Echinococcus* spp. prevalence in dog populations from two villages in Shiqu County from 2015 to 2017. Dog feces were collected, dietary fecal remains were checked, and the copro-PCR method was used to analyze the differences in *Echinococcus* spp. prevalence in dogs in the two villages and between different years. To evaluate the correlation between the implementation of the control project and *Echinococcus* spp. prevalence in dogs, questionnaire surveys were carried out, and their results were further compared with fecal sample analytic data. It is hoped that this study can help the evaluation of the effectiveness of dog management and intervention programme in Shiqu and benefit the long-term prevention and echinococcosis control in other pastoral areas of western China.

## Methods

### Study area

**Fig. 1:**
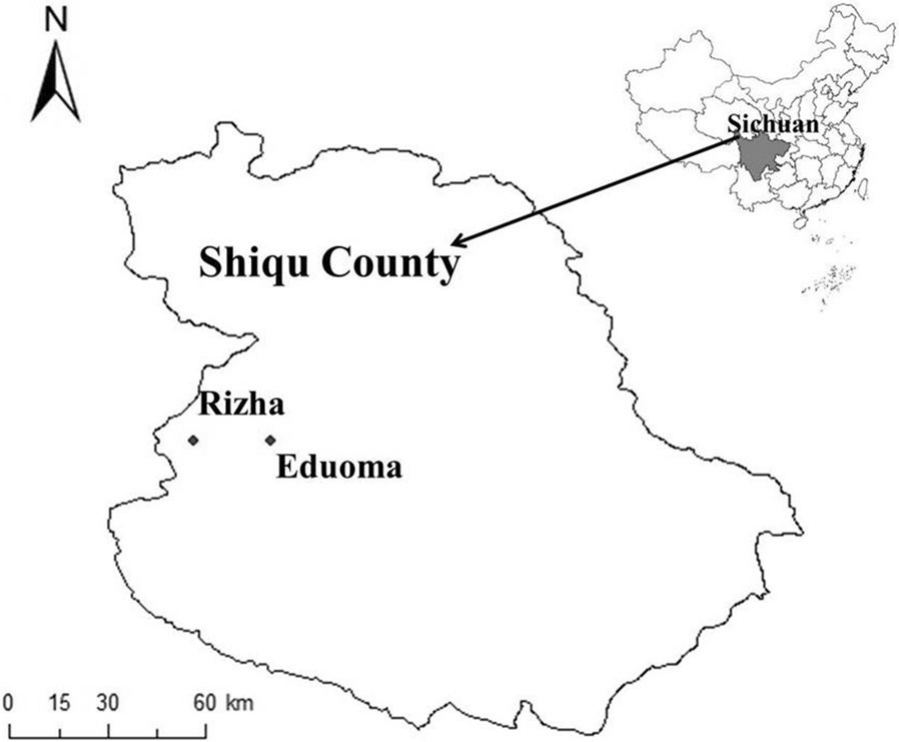
The geographical location of the study area, Shiqu County, in Sichuan, China

Studies were carried out in two villages, Eduoma (33°08′N, 97°47′E) and Rizha (33°07′N, 97°36′E) in Serxu Township (the former Eduoma Township, renamed in 2012), Shiqu County, which are located in the eastern Tibetan Plateau, Sichuan Province with an elevation between 4200–4700 m above sea level (Fig. 1). While Eduoma is the elementary education and primary health care center of Serxu Township with 160 houses, Rizha is a remote and smaller village with only 56 houses. All the houses in the two villages were newly built between 2014–2015 for nomadic Tibetan people supported by the central governmental settlement construction programme started in 2004. A local Tibetan family may occupy several houses in the village, due to a large number of family members. Rizha was surveyed in July and August 2015 and 2017, and Eduoma in July and August 2016 and 2017. Traditionally, summer is an important season for pastoralism in Shiqu County, when livestock herds need to graze on high elevation summer pasture areas distant from villages during late May to late September. Because many families have transitioned from nomadic to sedentary lifestyles, a cooperative pasturing relationship has been developed. Each year, only a small number of families or people in the village go to the summer pasture to keep livestock herds on behalf of the whole village and some of the owned dogs in the village are taken to summer pastures for shepherding, while families remaining in the village take care of the houses for owners working in summer pastures. Because of the more sedentary lifestyle, dogs are no longer considered absolutely necessary and as a result owned dog numbers in villages have decreased in recent years.

### Questionnaires

In each village, all households were visited. For each family with dogs, a questionnaire concerning information about dog keeping and echinococcosis prevention was given to adult family members. Dog keeping questions included the number of dogs owned, age and sex of each dog. More detailed information about echinococcosis prevention included three questions:

(i) Were dogs restrained from roaming during the past 12 months? Under the dog management regulations, all owned dogs should be leashed 24 h per day when staying in human settlements. Three kinds of dog keeping methods to restrain the dog roaming behavior in the village area: leashed (always tired up), night free (unleashed dogs at night but tired up in daytime), unleashed (always set free).
(ii) How frequently were dogs dewormed during the past 12 months? According to the regulation, each dog should be dosed by taking one pill containing 0.1 g praziquantel on the 10th day of each month after its distribution by local government staff who provide the anthelminthic to dog owners for free. Owners however may report to dose their dog (s) monthly, or irregularly (spanning from two to six months per dose), or never (did not dose during the past year).
(iii) What kind of food were owned dogs given during the past one year? Most dog owners would provide dogs with family meal leftovers of mainly roasted barley called Zamba, however, some owners would feed their dogs with livestock viscera when available.

### Dog fecal sample collection

When giving the questionnaire, dog fecal samples were collected from each household. Owned dogs were usually tied up separately, so one dog fecal sample for each owned dog could be collected around its normal tethering place/kennel. If the family owned several dogs and leash places were too near to distinguish feces origin, only one fecal sample was collected. During household visits, the rest of the village was checked to collect unleashed dog (owned or stray dogs) feces and to record numbers of unleashed dogs. All fecal samples were stored separately in screw 50 ml capped tubes with 95% ethanol. To kill any infective eggs of *Echinococcus* species, all fecal samples were stored at − 80 °C for at least one week before further processing [15].

### Fecal sample pre-treatment and copro-PCR

Fecal pre-treatment was carried out according to the method proposed by Jiang et al. [16]. Two to three grams of each dog fecal sample were suspended and stirred in 45 ml of deionized H_2_O (dH_2_O), incubated at 80 °C for 10 min, poured into a cell culture dish lined with double-layer medical sterilize gauze, and squeezed. The residue in the gauze was stored at 4 °C for dietary analysis. The squeezed suspension in the dish was placed in a 50 ml tube and centrifuged at 3600× *g* for 30 min. The supernatant was removed, and the sediment emulsified in 600 µl InhibitEx buffer (QIAamp Fast DNA stool mini kit; Qiagen, Hilden, Germany) and incubated at 70 °C for 10 min. The emulsified liquid was then transferred to a Precellys tube (Peqlab Biotechnology, Erlangen, Germany) and homogenized using an equal amount of Precellys ceramic beads with a diameter of 0.5 mm (Peqlab Biotechnology) in a Bertin Precellys 24 homogenizer (Bertin Technologies, Aix en Provence, France), at 5500× *rpm* for 15 s. This was repeated twice to achieve mechanical disruption of *Echinococcus* spp. eggs. The supernatant was collected for DNA extraction according to the instruction steps of the QIAamp Fast DNA stool mini kit.

Specific *nad*1 primers [17] were used to test the copro-DNA for *Echinococcus* species. All PCRs were performed in 25 μl volumes with 2 μl template DNA, 0.5 μl of the primers (10 μmol/l), 0.5 μl of bovine serum albumin (BSA; TaKaRa, Dalian, China), and 12.5 μl Premix Taq (Ex Taq Version 2.0 plus dye; TaKaRa), made up to a final volume of 25 μl with dH_2_O. The parameters of the PCRs for the three *nad*1 specific primer pairs were: 94 °C for 5 min, followed by 35 cycles of 94 °C for 30 s, 45 s at the annealing temperature of each primer pair (Table 1), 72 °C for 90 s, and a final extension step at 72 °C for 10 min. All PCRs were completed on a DNA thermal cycler (Applied Biosystems Veriti thermal cycler; Life Technologies, Carlsbad, CA USA). Negative controls (dH_2_O) were included in each PCR run.

**Table 1:** General information of copro-PCRs used in the study [13]

Primer	Species	Target gene	Sequence	Amplicon length (bp)	Annealing temperature (°C)
E.m	*E. multilocularis*	EmF19/3	TAGTTGTTGATGAAGCTTGTTG	207	53
		EmR6/1	ATCAACCATGAAAACACATATACAAC		
E.s	*E. shiquicus*	EsF50	TTATTCTCAGTCTCGTAAGGGTCCG	442	60
		EsR73	CAATAACCAACTACATCAATAATT		
E.g	*E. granulosus*	Eg1F81	GTTTTTGGCTGCCGCCAGAAC	226	62
		Eg1R83	AATTAATGGAAATAATAACAAACTTAATCAACAAT		

PCR products were subjected to agarose gel electrophoresis and stained with ethidium bromide (EB). Positive results indicated that the target gene fragments were amplified. Positive amplicons were excised carefully from the gel and purified with the TIAN gel Midi Purification Kit (Tiangen, Beijing, China). Cloning and sequencing of the purified products were conducted by Sangon Biotech Technology Co. Ltd. (Shanghai, China). Sequences were compared with the sequences on GenBank (http://www.ncbi.nlm.nih.gov/BLAST). Sequences with ≥ 99% identity were considered to represent specific *Echinococcus* species.

### Fecal sample food remnant analysis

Remains of each molecular analyzed dog fecal sample were checked for dietary items. The purpose of the food remnant analysis was to double check the questionnaire results and evaluate the possibility of owned dogs preying on intermediate hosts of *Echinococcus* spp., especially small mammal hosts of *E. multilocularis*. Checks were made for human food, especially roasted barley, and also the bones and teeth of small mammals such as pikas or voles. Before the analysis, the remains of each molecularly analyzed fecal sample were decontaminated by autoclaving in a wet atmosphere at 180 °C for 30 min and washed with water above a sieve (500 μm mesh size) to isolate the undigested food remnants.

### Statistics

We used the Chi-square test to compare the difference in the deworming frequencies and *Echinococcus* spp. copro-prevalence in dog populations between villages in different years based on data from the copro-DNA analysis and questionnaire. Although the sampling and questionnaire studies started in different years in the two villages, they were both resampled in 2017, two years after the announcement of the pilot echinococcosis control programme in 2015 [14]. The overall deworming rate and *Echinococcus* spp. copro-prevalence in the two villages in 2017 were used to compare with data collected in earlier years to evaluate the effectiveness of the control programme. It is important to note that since each village was sampled twice in different years, it is not certain whether the questionnaire and fecal samples collected from the same household were actually from the same family and the same dogs between years, due to the cooperative pasturing relationship mentioned earlier; therefore, data from different years for each household were not compared. All statistics were conducted using R 3.5.3 [18].

To evaluate the effect of the dog management and control programme on *Echinococcus* spp. prevalence in owned dog populations, logistic regression models were built. *Echinococcus* infection status of each owned dog according to copro-PCR results was the dependent variable by setting “not infected” and “infected” as the binary results. Eight variables based on the location and time of sampling and answers of the questions in the questionnaire were used as independent variables, including feeding habitats (human food and livestock viscera); sex of dogs (male and female); deworming frequency (monthly, irregular and never); village (Rizha and Eduoma); year of survey (2015, 2016 and 2017); number of dogs (owned and kept in each household); dog roaming behavior (leashed, night-free and unleashed) and the age of the dog. Considering the independence requirement of each dog sampled, to the household with multiple dogs, information from only one randomly chosen dog was imported into the model.

The logistic regression model significance level was set to *P* < 0.05. The coefficient of determination of the final model was expressed by the Nagelkerke *R*^2^ [19]. The logistic regression model analysis was conducted using SPSS 23.0 (IBM, 2015).

## Results

During the entire research period, 69 households covering 318 people (owners of 84 dogs) were questioned and 105 dog fecal samples were collected from 75 from owned dogs and 30 from unleashed dogs (Table 2). Seven unleashed dogs were observed in Rizha Village in 2015 and one unleashed dog was recorded in Eduoma Village in 2016. No unleashed dogs were observed in either village in 2017.

**Table 2:** Summary of questionnaires and fecal sample collection in Rizha and Eduoma villages, Shiqu County, Sichuan, China, between 2015–2017

Year	Village	No. of questionnaires	No. of dogs recorded		No. of fecal samples collected	
			Owned	Unleashed	Owned	Unleashed
2015	Rizha	11	14	7	14	27
2016	Eduoma	27	31	1	25	1
2017	Rizha	12	16	0	14	1
2017	Eduoma	19	23	0	22	1
Total		69	84	8	75	30

### Questionnaire

#### Dog ownership, sex and age distribution

**Fig. 2:**
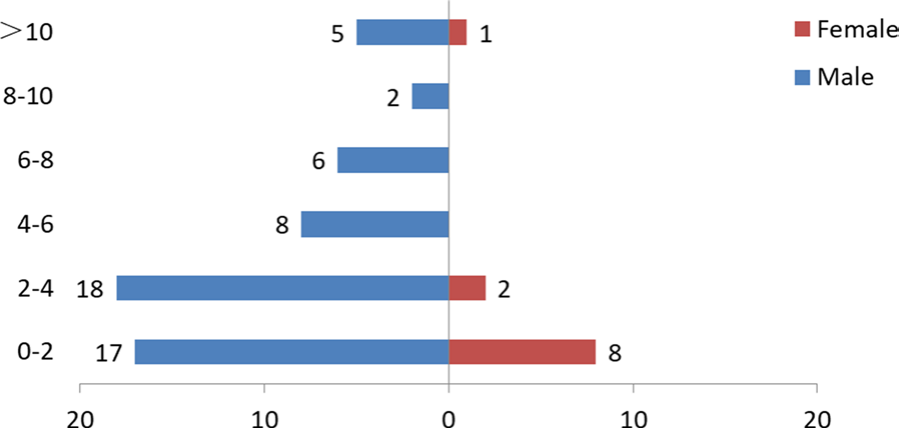
A population pyramid for owned dogs sampled in Rizha and Eduoma villages (*n* = 67)

No household was recorded keeping more than two dogs in the two villages during the entire study period (Table 3), with a mean number of 1.2 ± 0.42 (± SD) dogs kept in each of the 69 visited households. There was no significant difference in the number of dogs kept per household detected between the two villages and between years (Rizha 2015 *vs* 2017, *χ*^2^ = 0.007, *df* = 1, *P* = 0.933; Eduoma 2016 *vs* 2017, *χ*^2^ = 0.017, *df* = 1, *P* = 0.896). Most dogs owned by pastoralists were male (83.1%) (Table 3). Among the 84 recorded dogs, information of sex and age was matched in 67 individuals and ages were mainly 4 years-old or younger (67.2%) (Fig. 2), ranging from less than one year to more than 10 years-old.

**Table 3:** Summary of 69 questionnaires from Rizha and Eduoma villages, Shiqu County, Sichuan, China, between 2015–2017

Subjects in the questionnaire	Sampling village	Dog status	No. of owned dogs (No. of households)		
			2015	2016	2017
Number of dogs recorded (*n* = 84)	Rizha		14 (11)	–	16 (12)
	Eduoma		–	31 (27)	23 (19)
Sex (*n* = 71)	Rizha	Male	11 (10)		13 (11)
		Female	1 (1)		1 (1)
	Eduoma	Male		20 (20)	15 (14)
		Female		7 (7)	3 (3)
Roaming behavior (*n* = 73)	Rizha	Leashed	13 (10)	–	14 (12)
		Night free	1 (1)	–	0
		Unleashed	0	–	0
	Eduoma	Leashed	–	21 (21)	20 (18)
		Night free	–	4 (4)	0
		Unleashed	–	0	0
Deworming frequency (*n* = 67)	Rizha	Monthly	–	–	13 (11)
		Irregular	5 (4)	–	1 (1)
		Never	6 (5)	–	0
	Eduoma	Monthly	–	23 (23)	17 (15)
		Irregular	–	2 (2)	0
		Never	–	0	0
Feeding habits (*n* = 84)	Rizha	Human food	14 (11)	–	16 (12)
		Viscera	0	–	0
	Eduoma	Human food	–	31 (27)	23 (19)
		Viscera	–	5 (3)	4 (3)

*Abbreviation*: n, number of owned dogs recorded

#### Dog roaming behavior

Most of the recorded dogs in the two villages (93.2%) were kept leashed and the proportion of dogs kept leashed did not differ between villages and years (Table 3). No all-day unleashed owned dogs were recorded during the entire research period. Night-free dogs were reported from one Rizha household (7.1%) in 2015 and four Eduoma households (16%) in 2016 (Table 3). In 2017, 100% of the households in Rizha and Eduoma that responded to the questionnaire, kept their dogs leashed 24 h per day. No significant *χ*^2^ analysis results of the difference in the dog roaming behavior (i.e. leashed/night free) were detected between years and villages, respectively: (i) Rizha 2015 *vs* 2017, *χ*^2^ = 0.019, *df* = 1, *P* = 0.891; (ii) Eduoma 2016 *vs* 2017, *χ*^2^ = 0.162, *df* = 1, *P* = 0.687; (iii) Rizha *vs* Eduoma in 2017, *χ*^2^ = 0.022, *df* = 1, *P* = 0.882.

#### Deworming frequency and feeding behavior

The dog monthly deworming rate was 93% in Rizha in 2017, according to the responses from visited households (Table 3). However, in 2015 no dogs in Rizha were reported to be dewormed monthly during the past year (Table 3). In addition the deworming frequency (i.e. monthly/(irregular + never)) was significantly different among years for Rizha (2015 *vs* 2017, *χ*^2^ = 5.850, *df* = 1, *P* = 0.016). In Eduoma, the monthly deworming rates were always high and there was no significant difference between the rates in 2016 and 2017 (*χ*^2^ = 0.035, *df* = 1, *P* = 0.853). There was no significant difference in the rate of dogs monthly deworming between Rizha and Eduoma in 2017 (*χ*^2^ = 0.021, *df* = 1, *P* = 0.886)

All households in Rizha in 2015 and 2017 responded that they fed dogs with family left-over food, especially roasted barley. However, in Eduoma in 2016 three households owning five dogs, and in 2017 three households owning four dogs, responded that they also fed their dogs livestock viscera when available (Table 3).

### Copro-DNA *Echinococcus* spp. prevalence in dogs

All 105 fecal samples were tested by *Echinococcus* copro-PCR analysis. In general, the overall *Echinococcus* copro-prevalence in dogs in Rizha decreased significantly from 51.2% in 2015 to 20.0% in 2017 (*χ*^2^ = 6.850, *df* = 1, *P* = 0.009), while the prevalence in Eduoma was low at 11.5% in 2016, 4.3% in 2017, and not significantly different between the two sampling years (*χ*^2^ = 0.842, *df* = 1, *P* = 0.359) (Table 4). Although the overall *Echinococcus* prevalence in the first sampling year in Rizha (2015) was significantly higher than that in Eduoma (2016) (*χ*^2^ = 4.485, *df* = 1, *P* = 0.034), the difference in prevalence was not significant in 2017 (*χ*^2^ = 0.700, *df* = 1, *P* = 0.404).

**Table 4:** Statistics of the *Echinococcus* copro-DNA prevalence in owned and unleashed dogs in Rizha and Eduoma villages, Shiqu County, Sichuan, China, between 2015–2017

Village	Year	Dog status	*E. multilocularis*	*E. shiquicus*	*E. granulosus*	*Echinococcus* spp.
Rizha	2015	Owned	50.0 (26.8–73.2) (7/14)	28.6 (9.6–58.0) (4/14)^a^	0 (0/14)	50.0 (26.8–73.2) (7/14)
		Unleashed	48.1 (29.2–67.6) (13/27)	40.7 (23.0–61.0) (11/27)^b^	3.7 (0.1–20.9) (1/27)^c^	51.9 (32.4–70.8) (14/27)
		Sub-total	48.8 (33.2–64.6) (20/41)	36.6 (22.6–53.1) (15/41)	2.4 (1.3–14.4) (1/41)	51.2 (35.4–66.8) (21/41)
Eduoma	2016	Owned	4.00 (0.2–22.3) (1/25)	0 (0/25)	4.0 (0.2–22.3) (1/25)	8.0 (1.4–27.5) (2/25)
		Unleashed	0 (0/1)	0 (0/1)	– (1/1)	– (1/1)
		Sub-total	3.8 (20.2–21.6) (1/26)	0 (0/26)	7.7 (1.3–26.6) (2/26)	11.5 (3.0–31.3) (3/26)
Rizha	2017	Owned	21.4 (5.7–51.2) (3/14)	0 (0/14)	0 (0/14)	21.4 (5.7–51.2) (3/14)
		Unleashed	0 (0/1)	0 (0/1)	0 (0/1)	0 (0/1)
		Sub-total	20 (5.3–48.6) (3/15)	0 (0/15)	0 (0/15)	20 (5.3–48.6) (3/15)
Eduoma	2017	Owned	4.6 (0.1–9.0) (1/22)	0 (0/22)	0 (0/22)	4.6 (0.1–9.0) (1/22)
		Unleashed	0 (0/1)	0 (0/1)	0 (0/1)	0 (0/1)
		Sub-total	4.3 (0.2–24.0) (1/23)	0 (0/23)	0 (0/23)	4.3 (0.2–24.0) (1/23)
Rizha and Eduoma	2017	Owned total	11.1 (3.6–27.0) (4/36)	0 (0/36)	0 (0/36)	11.1 (3.6–27.0) (4/36)
		Total	10.5 (3.4–25.7) (4/38)	0 (0/38)	0 (0/38)	10.5 (3.4–25.7) (4/38)

^a^Four owned dogs were detected mixed infection of *E. multilocularis* and *E. shiquicus*

^b^Ten unleashed dog fecal samples were detected with mixed infection of *E. multilocularis* and *E. shiquicus*

^c^One unleashed dog fecal sample was detected with mixed infection of *E. granulosus*, *E. multilocularis* and *E. shiquicus*

*Note:* Data are presented as follows: prevalence (95% CI) in % (number of positive fecal samples/ total number of fecal samples examined)

DNA sequences of *E. multilocularis*, *E. shiquicus* and *E. granulosus* were all detected in dog fecal samples by copro-PCR analysis. *Echinococcus multilocularis* and *E. shiquicus* were the main *Echinococcus* species detected in Rizha dog fecal samples in 2015 (Table 4), and the prevalence of these two species were not significantly different (*χ*^2^ = 0.502, *df* = 1, *P* = 0.479). Meanwhile, the prevalence of *E. multilocularis* and *E. shiquicus* were both significantly higher than that of *E. granulosus* (*E. multilocularis vs E. granulosus*, *χ*^2^ = 14.168, *df* = 1, *P* < 0.001; *E. shiquicus vs E. granulosus*, *χ*^2^ = 10.464, *P* < 0.001). However, *E. multilocularis* was the only *Echinococcus* species constantly detected in owned dogs in both villages during the entire study period, although the prevalence declined (Table 4). *Echinococcus shiquicus* was only detected in dogs in Rizha in 2015 and *E. granulosus* was only detected from two unleashed and one owned dog fecal samples in the two villages during the first sampling years, while both *E. granulosus* and *E. shiquicus* not detected in 2017 (Table 4). Feces of unleashed dogs were mainly collected in Rizha in 2015 (Table 2) and the copro-prevalence of the three *Echinococcus* species could be as high as or even higher than those of owned dogs in the same year (Table 4), although differences were not significant (*χ*^2^ = 0.013, *df* = 1, *P* = 0.910).

### Fecal food remnant analysis

Most of the dog feces contained remains of roasted barley (97.1%). Small mammal teeth and bones were only found in three dog fecal samples, including one unleashed dog fecal sample from Rizha in 2015, one owned dog fecal sample from Rizha in 2016 and one owned dog fecal sample from Eduoma in 2017.

### Significant variables related to the prevalence of *Echinococcus* in owned dogs

Among the 75 fecal samples from owned dogs, data from 59 qualified to enter the logistic regression model. The final model revealed that three significant variables influencing the *Echinococcus* infection of owned dogs in villages were the deworming frequency, sex of dogs and their feeding habits (Nagelkerke *R*^2^ = 0.492, Table 5). Monthly deworming seemed to be vital to reduce the prevalence of canine echinococcosis, because irregular or no deworming could resulted in the infection odds ratio (OR) to increase from 23.3 to 52.5 times (Table 5). *Echinococcus* spp. prevalence showed a significant sex bias in dogs with male dogs having infection risks more than 1000 times higher than female dogs (Table 5). Feeding dogs with livestock viscera was the third statistically significant risk causing *Echinococcus* spp. prevalence to increase (OR = 23.3, *P* = 0.021). Feeding dogs with livestock viscera is judged as a main risk of *E. granulosus* infection. However, since only two fecal samples with *E. multilocularis* infection and no *E. granulosus* infection were detected from the six fecal samples from households reporting feeding dogs on viscera, no further analysis of the feeding habits variable using logistic regression models was carried out.

**Table 5:** Binary logistic regression analysis of significant variables associated with *Echinococcus* spp. infections in owned dogs

Variable	State	Infection test		*P*-value	Odds ratio
		Infected	Uninfected		
Deworming frequency	Monthly	3	45	0.010	1
	Never	2	3	0.021	23.3
	Irregular	3	3	0.004	52.5
Sex	Female	0	8	–	1
	Male	8	43	0.001	> 1000

## Discussion

The huge pastoral area of the eastern Tibetan Plateau, as typically represented by Shiqu County (Sichuan Province), has been recognized as one of the most serious echinococcosis endemic regions in the world [8, 20]. Thus, Shiqu has been listed by the Chinese government as a pilot area of the national programme for prevention and control of echinococcosis [21]. The increasing prevention awareness of echinococcosis in Tibetan communities has resulted in a better implementation of dog population management, regular deworming and changes to better dog feeding. As an important part of the pilot project, the dog management work has significantly reduced the prevalence of canine echinococcosis since its implementation in 2015 (Table 5).

According to the NDRC in 2016 [7], the population size of stray dogs must be controlled and decreased and the canine echinococcosis prevalence in dog populations should be less than 5% in endemic areas by the end of 2020. The dog management regulations were strictly implemented in Rizha right after the beginning of the Shiqu county-wide implementation of the echinococcosis prevention and control programme in November 2015 [14]. The questionnaire and copro-PCR results showed very high *Echinococcus* spp. prevalence (51.2%) in dogs in Rizha in the summer of 2015, which was significantly decreased (20%) by 2017, two years after the implementation of the dog management regulations (Table 4). By contrast, a trial of the dog management regulations has been carried out in Eduoma Village since 2014, earlier than the county-wide implementation, thus the low *Echinococcus* spp. prevalence in Eduoma recorded in 2016 and 2017 (Table 4) was not unexpected. The overall *Echinococcus* spp. prevalence in owned dogs in Eduoma was less than 5% in 2017 (Table 4), which met the standard baseline defined by NDRC [7] and an obvious decreasing trend in *Echinococcus* spp. prevalence in the dog population was confirmed by our current surveillance data.

The importance of dog population control for echinococcosis has been studied in detail by some reports [20, 22]. Although the regulations of the dog management programme have been implemented in Shiqu County for many years [23, 24], it needs time to cover all the remote areas of the county. For example, numbers of owned dogs per household of the two target villages were less than two on average which was below the number permitted by the dog management regulations, and furthermore dog ownership was not different between villages or between sampling years. However, stray dog populations were different (Table 2). In Eduoma, the dog population has been strictly controlled since 2014, and presence of unleashed dogs was unusual during the entire sampling period of this study (Table 2). In the more remote Rizha, unleashed dogs were still common place in 2015, and it was where the majority of the unleashed dog feces and unleashed dogs in this study were recorded (Table 2). Numbers of unleashed dog associated feces significantly decreased in 2017 and in that years unleashed dogs were not observed (Table 2). Although dog feces might also have come from unleashed owned dogs, judging by the high rate of awareness of echinococcosis control and prevention in the community (Table 3) and the reduction in numbers of unleashed dogs and ground feces collected, this probably reflected improved control of the unowned stray dog population. In general, the present data indicated that the dog management measures did significantly reduce *Echinococcus* spp. prevalence in local populations of dogs in some villages in Shiqu County and should be sustained for effective echinococcosis control in pastoral Tibetan communities.

All of the three *Echinococcus* spp. reported in China were detected infecting dogs in our study (Table 4). *Echinococcus granulosus* and *E. multilocularis* are the two confirmed zoonotic species. The overall prevalence of *E. multilocularis* was significantly higher than that of *E. granulosus* in dogs in our study (Table 4) and this was similar to previous observations [11, 25]. The prevalence of *E. shiquicus* copro-DNA could be as high as *E. multilocularis* in dog feces (Table 4), which further supported the possibility of dogs as a viable definitive host species of *E. shiquicus*, as suggested by Boufana et al. [13]. Although no transmission to humans has been reported, *E. shiquicus* shares a sylvatic transmission cycle with *E. multilocularis* between canids and small mammal species [13, 26, 27]. *Echinococcus multilocularis* was the main and the only *Echinococcus* species continuously detected in dogs, especially in owned dogs in the two visited villages in all sampling years (Table 4). Compared with preventing owned dogs from becoming infected with *E. granulosus* by ingesting livestock viscera, it would seem more difficult to stop the trophic connection between owned dogs and small mammals to prevent infection with *E. multilocularis.* Factors influencing the *Echinococcus* spp. prevalence in owned dogs are important for dog targeted control measures in the pastoral areas of the Tibetan Plateau.

Free roaming has been considered a significant risk for infections of owned dogs with *E. multilocularis* and *E. shiquicus*, but not with *E. granulosus* in Shiqu County because of their high chances of contact with and preying upon wild intermediate host small mammals [11, 25]. Unleashed dogs can be active within and around Tibetan villages, as shown by Vaniscotte et al. [12] who reported that a released owned dog could move up to 1500 m away from the village with an average activity area of 77 ± 59.4 ha. Such an active spatial behavior pattern enables a free-roaming dog to visit areas where wild small mammal intermediate host species may be distributed. The average worm lifespan of *E. granulosus* and *E. multilocularis* is probably ten and five months respectively [1], so theoretically preventing contact with intermediate hosts, deworming an infected dog and restraining roaming behavior are considered as effective methods to control *Echinococcus* spp. prevalence in owned dogs [7]. Because almost all households from the two Tibetan villages claimed to leash their dogs 24 hours per day according to questionnaire results, there were not enough negative samples to result in the dog roaming behavior being assessed as an insignificant variable by the logistic regression analysis. The questionnaire results suggested however that, as an important part of health education, leashing dogs had been generally accepted and followed by the two communities.

However, the fact that copro-DNA of *E. multilocularis* was continually detected in owned dogs indicated that owned dogs still have chances to come into contact with small mammal host species. Small mammal bones were detected in feces of a few owned dogs and a minority of households released dogs at night (Table 3). Even if people leashed dogs as instructed, leashed dogs may also be able to prey on peri-domestic small mammals. At least six widespread small mammal species, mainly voles and pikas have been identified in Shiqu County as intermediate hosts of *E. multilocularis* [20] and the prevalence in voles species was significantly higher than that in pikas [23, 28, 29]. Mu [30] confirmed that the population density of small mammals especially vole species can be high less than 500 m away from Rizha Village. In fact, evidence of small mammal presence could be as near as 32 m away from households in villages of Shiqu County [12]. Therefore, infected small mammals may have the opportunity to access the villages, which may provide leashed dogs opportunities to prey upon them. Moreover, when herding on the summer pasture, owned dogs are usually unleashed all the time and these dogs could be infected by preying on small mammals before they come back to the village. Therefore, although restraining dogs is considered a fundamental measure to decrease *Echinococcus* spp. prevalence in owned dogs, proactive measures such as regular dosing with praziquantel are still needed.

Regular supervised dog praziquantel dosing has been considered to be the pivotal measure for echinococcosis control and prevention in the pastoral areas of the Tibetan Plateau [6], beginning in northwest Sichuan Province in 2006 [31]. The logistic regression model revealed that monthly dosing was significantly more powerful than irregular or no dosing to decrease *Echinococcus* spp. prevalence in owned dogs (Table 5). Protoscoleces of *E. multilocularis* and *E. granulosus* (*s.s*.) usually need four to six weeks to respectively develop into adult tapeworms after infection [1], so monthly dog deworming has been adopted as the most important control measure.

However, the application of monthly dog dosing in remote settled and semi-nomadic Tibetan communities is challenging. In the more remote Rizha Village, none of the owned dogs surveyed were monthly dosed in 2015 and one household stated in 2017 that they had not received any praziquantel for more than half a year. Because of the obvious difficulty in seasonal traffic transport restrictions, communications and the highly mobile semi-nomadic Tibetan lifestyles (despite permanent settlements being provided), administration of monthly dosing in remote communities is still difficult to enforce in all families. Although monthly dosing regulation can be effectively supervised in some settlements as demonstrated by our data in 2016 and 2017, the effectiveness of monthly dog dosing cannot be easily supervised in summer pasture areas where nomadic families scatter on the vast high-altitude grasslands. This emphasizes the extreme importance of supervised monthly dosing of owned dogs when semi-nomadic families gather in permanent settlements in villages from late September to late May.

Long-term supervised dog dosing programmes can be extremely costly and resource-demanding. The several successful applications of regular praziquantel dosing were usually associated with *E. granulosus* control in more developed agricultural areas [32–34]. In those regions *E. granulosus* is mainly transmitted between large herbivorous livestock and dogs, therefore coupled with livestock slaughter and viscera management, long term dog dosing can result in a significant impact to the transmission cycle [35]. With regards to the more pathogenic *E. multilocularis* in the co-endemic regions of eastern Tibetan Plateau, more complex wildlife transmission cycles are present involving wild canids such as the Tibetan fox (*Vulpes ferrilata*) and the red fox (*V. vulpes*) [16] and small mammals, such as pikas and small rodent species [27]. The large populations and dispersed distribution of small mammals [27, 36, 37], and their potential predation by dogs (even potentially when leashed), suggests that the possibility of *E. multilocularis* spreading from the wildlife reservoir to the human environment always exists. Once a regular dosing programme stops, *Echinococcus* spp. prevalence in dogs can return to pre-treatment levels in less than ten months [25], so in order to keep up the long-term regular dog dosing programme to cover the vast western pastoral areas of China, He et al. [28] suggested decreasing the dosing frequency from once per month to once per every two or three months as recommended by the WHO [38]. Their recommendation referred to dog re-infection studies of *E. granulosus*, however not enough empirical data regarding *E. multilocularis* re-infection in Tibetan dogs are available yet. Our study suggests that interruption of dosing for several months can significantly decrease the power of the praziquantel dosing. Because of its shorter prepatent infection period and more complex transmission cycles, compared with *E. granulosus*, successful interruption of transmission of *E. multilocularis* with consideration of public health and economic feasibility still requires applied research.

A single dose of praziquantel is recommended to be 5 mg/kg for dogs [1]. At present, the one-dog-one-pill (0.1 g praziquantel) dosage per month neglects individual weight differences among dogs. Tibetans frequently expressed their concerns about the potential negative side effects of the drug during surveys in villages. The possible use of slow release praziquantel [39] may be a better choice for future large-scale implementation though such formulations require further assessment.

Dogs being male and older are two potential significant factors associated with higher *Echinococcus* spp. infection [11]. The significant effect of dogs being male was detected by the logistic regression model in this study. Traditionally, male dogs are preferred by Tibetan pastoralists for both better property and livestock protection and easier dog population management in the community. Because of the development of new settlements and the cooperative pasturing in nomadic local communities, people no longer need as many dogs as before. Controlling breeding activities by keeping only male dogs is usually one of the most feasible methods for remote and developing areas [22]. The proportion of male dogs is significantly higher than female dogs in local communities of Shiqu County in the present and previous studies [25, 40]. Compared with female dogs, male dogs are more likely to maintain territories and hunt, increasing the chances of infection. However, male territorial behavior and hunting are only important when dogs are unleashed. Therefore, if dogs were tied up well as reported by the most visited households, the fact that all canine echinococcosis infections were detected in male dogs in this study should be the result of male dogs being the majority (Table 5) but not a significant infection risk. As to the age bias, the infection burden of *E. granulosus* could be significantly lower in dogs over five years-old, but not significant for *E. multilocularis* infection [11, 20]. In the present study, the fact that most of sampled dogs were less than five years-old and *E. multilocularis* was the main *Echinococcus* species but not *E. granulosus* (Table 5) might explain the insignificant effect of dog age.

Not feeding dogs with livestock viscera has frequently been recommended as one of the most effective methods to control domestic dogs infecting *E. granulosus* [41–43]. Since we did not detect *E. granulosus* infection in the fecal samples from viscera-fed dogs, the importance of dog feeding habits cannot be evaluated directly by the logistic regression model analysis. Nevertheless, the importance of not feeding dogs with livestock viscera is still significant in this study. In fact, most visited households reported being aware of and did not feed dogs with viscera (Table 3), and the prevalence of *E. granuluosus* decreased dramatically and was not detected in both villages in the last sampling year of the study (Table 4). All of these results suggest that the regulation to stop feeding dogs with livestock viscera has been well proceeded in local Tibetan communities and received expected effect.

## Conclusions

This study confirmed that, as a crucial part of the Chinese echinococcosis prevention and control project, the current dog management programme has significantly decreased the unowned dog population size and the prevalence of canine echinococcosis in dogs in two Tibetan villages of Shiqu County, Sichuan Province. Supervised monthly dosing with praziquantel was the most important method to reduce copro-prevalence of canine echinococcosis in owned dogs. Additionally, leashing dogs all the time in villages and avoidance of dogs feeding on livestock viscera were significant control measures. The sex and age of dogs may not be significant risks in the two villages, but the potential contact between leashed dogs and infected small mammals is worthy of special attention. Although only a small number of dogs were taken to summer pastures from the villages, the infection dynamics of these dogs remains undetermined. This study also confirmed the presence of *E. shiquicus* DNA in dog feces. Although significant reductions in canine echinococcosis prevalence were detected, the long-term application of regular dog dosing in the vast remote endemic areas of west China remains challenging and further data are required on optimal dosing frequency from these co-endemic areas.

## Data Availability

All data generated or analyzed during this study are included in this published article. The newly generated sequences of *Echinococcus shiquicus* were submitted to the GenBank database under the accession numbers MT259952-MT259957. No new haplotypes of *E. multilocularis* and *E. granulosus* were generated in this study.

## Abbreviations

AE: alveolar echinococcosis
CE: cystic echinococcosis
SD: standard deviation
*nad*1: NADH dehydrogenase subunit 1 gene
NDRC: National Development and Reform Commission
WHO: World Health Organization
