# Correction to: The effects of dog management on *Echinococcus* spp. prevalence in villages on the eastern Tibetan Plateau, China

**DOI:** 10.1186/s13071-021-04637-1

**Published:** 2021-02-26

**Authors:** Xiaodong Weng, Zhiqiang Mu, Xu Wei, Xu Wang, Qingqiu Zuo, Shuo Ma, Youzhong Ding, Xiaoming Wang, Weiping Wu, Philip S. Craig, Zhenghuan Wang

**Affiliations:** 1 School of Life Sciences, East China Normal University, Shanghai, China; 2 National Institute of Parasitic Diseases, Chinese Center for Disease Control and Prevention, Shanghai, China; 3 Shanghai Science and Technology Museum, Shanghai, China; 4 Joint Translational Science & Technology Research Institute, East China Normal University, Shanghai, China; 5 School of Environment and Life Sciences, University of Salford, Greater Manchester, UK; 6 Shanghai Key Laboratory of Urbanization and Ecological Restoration, East China Normal University, Shanghai, China

## Correction to: Parasites Vectors (2020) 13:207 https://doi.org/10.1186/s13071-020-04082-6

Following publication of the original article [1], the authors flagged two errors in the Discussion:

The sentence “Dogs being male and older are two potential significant factors associated with higher *Echinococcus* spp. infection [11]” had been incorrectly written as “Dogs being male and older are two significant factors associated with higher *Echinococcus* spp. infection [11]”;

While the sentence “As to the age bias, the infection burden of *E. granulosus* could be significantly lower in dogs over five years-old, but not significant for *E. multilocularis* infection [11, 20]” had been given incorrectly as “As to the age bias, the infection burden of *E. granulosus* could be significantly higher in dogs over five years-old, but not significant for *E. multilocularis* infection [11, 20]”.

These errors have now been published in the original article.

The authors apologize for any inconvenience caused.
